# Accessing Artificial Intelligence for Fetus Health Status Using Hybrid Deep Learning Algorithm (AlexNet-SVM) on Cardiotocographic Data

**DOI:** 10.3390/s22145103

**Published:** 2022-07-07

**Authors:** Nadia Muhammad Hussain, Ateeq Ur Rehman, Mohamed Tahar Ben Othman, Junaid Zafar, Haroon Zafar, Habib Hamam

**Affiliations:** 1 Lambe Institute for Translational Research, National University of Ireland Galway, H91TK33 Galway, Ireland; n.muhammadhussain1@nuigalway.ie (N.M.H.); haroon.zafar@nuigalway.ie (H.Z.); 2 College of Engineering and Informatics, National University of Ireland Galway, H91TK33 Galway, Ireland; 3 Department of Electrical Engineering, Government College University, Lahore 54000, Pakistan; ateqrehman@gmail.com (A.U.R.); chairperson.engineering@gcu.edu.pk (J.Z.); 4 Department of Computer Science, College of Computer, Qassim University, Buraydah 51452, Saudi Arabia; 5 Cardiovascular Research and Innovation Centre Ireland, School of Medicine, National University of Ireland Galway, H91TK33 Galway, Ireland; 6 Faculty of Engineering, Université de Moncton, Moncton, NB E1A3E9, Canada; habib.hamam@umoncton.ca; 7 International Institute of Technology and Management, Commune d’Akanda, Libreville 1989, Gabon; 8 Spectrum of Knowledge Production and Skills Development, Sfax 3027, Tunisia; 9 Department of Electrical and Electronic Engineering Science, School of Electrical Engineering, University of Johannesburg, Johannesburg 2006, South Africa

**Keywords:** fetus classification, deep neural networks, transfer learning, cardiotocography, artificial intelligence, clinical settings

## Abstract

Artificial intelligence is serving as an impetus in digital health, clinical support, and health informatics for an informed patient’s outcome. Previous studies only consider classification accuracies of cardiotocographic (CTG) datasets and disregard computational time, which is a relevant parameter in a clinical environment. This paper proposes a modified deep neural algorithm to classify untapped pathological and suspicious CTG recordings with the desired time complexity. In our newly developed classification algorithm, AlexNet architecture is merged with support vector machines (SVMs) at the fully connected layers to reduce time complexity. We used an open-source UCI (Machine Learning Repository) dataset of cardiotocographic (CTG) recordings. We divided 2126 CTG recordings into 3 classes (Normal, Pathological, and Suspected), including 23 attributes that were dynamically programmed and fed to our algorithm. We employed a deep transfer learning (TL) mechanism to transfer prelearned features to our model. To reduce time complexity, we implemented a strategy wherein layers in the convolutional base were partially trained to leave others in the frozen states. We used an ADAM optimizer for the optimization of hyperparameters. The presented algorithm also outperforms the leading architectures (RCNNs, ResNet, DenseNet, and GoogleNet) with respect to real-time accuracies, sensitivities, and specificities of 99.72%, 96.67%, and 99.6%, respectively, making it a viable candidate for clinical settings after real-time validation.

## 1. Introduction

Artificial intelligence (AI) is fueling and reshaping various aspects of healthcare, from personalized treatments to improved diagnostics [1]. Advances in health informatics and deep learning (a subset of AI) algorithms allow for modelling, which creates informed and improved health decision outcomes [2,3]. Deep neural networks (DNNs) are continuously exploring avenues with tangible impact in real-world clinical systems. DNNs are being deployed in various decision-support bio-medical systems including fetus classification. These are used to determine the compromised fetal status [4,5,6] to avoid hypoxic injury and pregnancy-related complications [7]. Cardiotocograms (CTGs) contain imperative information with respect to fetal heart rate (FHR), uterine contraction (UC) based on the fetus’s acceleration, deceleration, baseline heart rate, and heart rate variability. These parameters indicate the fetus’s hypoxic status and serve as a baseline for medical interventions. The complex CTG patterns are poorly understood and their visual interpretation by clinicians is challenging [8]. It is now well understood that the linear features in the CTG datasets have a more pronounced effect than the nonlinear ones in the modelling of fetuses [9]. Hence, the feature selection algorithms for CTG patterns allow dimensionality reduction with a slight compromise on the sensitivity and selectivity parameters [10]. Contrary to conventional machine learning (ML) approaches, convolutional neural networks (CNNs) do not require the execution of complex feature engineering. DNN models can self-learn useful features from the input data without compromising informative features.

An intercomparison of seven algorithms, including artificial neural networks (ANNs), long short-term memory networks (LSTMs), and random forests are reported elsewhere [11,12] on the CTG datasets, but did not accomplish the desired classification response in predicting the suspicious fetus state. This is attributed to the complexity of fetus dynamics and a considerable false-positive rate as indicated in previous studies [13,14]. Feedforward, multimodal and extreme learning networks (ELNs) are data-driven approaches. However, all these studies provide limited information on the effect of hyperparameters on the task of embryo morphological assessments [15,16]. DNNs worked effectively with persistent data using moving filters and max-pooling operations [16]. However, training DNNs for a converged solution is both time- and space-intensive and impedes their real-time implementation in clinical settings [17,18,19,20,21,22]. The resolve of this paper is to make progress toward a real-time clinical support system for all classes of CTG recordings.

Our CTG datasets consisted of 2126 data on pregnant women that contained 23 attributes related to FHR and UC. To process this large number of attributes in time-constrained settings, we proposed a time-efficient SVM-merged AlexNet classifier. SVMs are added at the fully connected layers of the AlexNet for the faster convergence of the hyperplane. Rather than learning from scratch, we partially froze early layers of the architecture and fine-tuned the learned features through the transfer learning technique [23,24,25]. We implemented our architecture, and an intercomparison was made with the leading works reported so far. The proposed architecture recorded the best classification performance in minimal time compared to other leading architectures making it an evidence-based choice in time-constrained settings. The proposed algorithm would help in realizing the development of better AI solutions for maternal–fetal upkeep.

The contributions of this paper may be summarized as follows:

1. By using SVM-merged DNNs on the CTG dataset, we achieved a faster convergence of the hyperplane, resulting in clinically relevant time performance. DNN automatically extracts features, and the generalized ability of SVMs was exploited for multiclass classification.
2. We exploited transfer learning to improvise classification speed by bypassing the training time of the data samples.
3. With the emergence of machine learning operations (MLOps), we presented a computationally lightweight model to achieve low latency in real-time settings.
4. Our model outperforms the leading algorithms with respect to fetus classification accuracy.

The paper is organized as follows: Section 2 describes the materials and methods used in this work. It encompasses details regarding the dataset and its preprocessing. Then, our proposed classification architecture is detailed with respect to hyperparameters and optimization of the cross-entropy-based loss function. Section 3 is devoted to results and analysis. Section 4 contains the related discussion on the work under consideration. Finally, Section 5 is the concluding section.

## 2. Materials and Methods

### 2.1. CTG Dataset and Preprocessing

**Figure 1:**
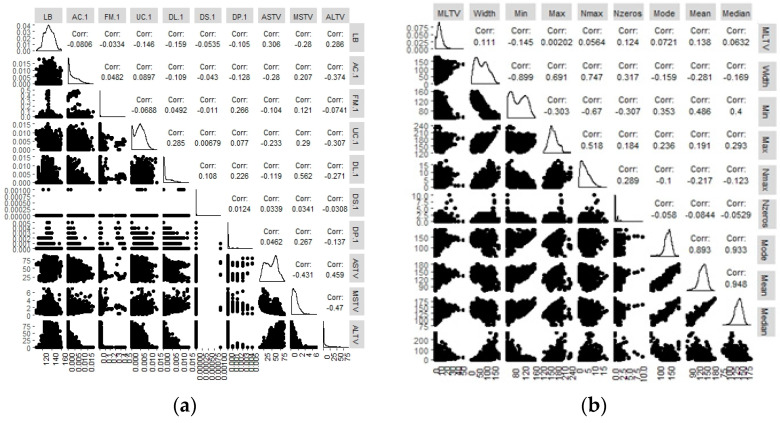
A depiction of correlation among different attributes of the CTG dataset, including LB, AC, FM, UC, DL, DS, ASTV, MSTV, and ALTV, in addition to min, max, width mode, mean and median of the FHR histogram.

In this study, the dataset used was obtained from the University of California Irvine Machine Learning Repository, which is a public dataset and is available for review [20]. It consisted of data from 2126 pregnant women. This dataset contains 23 attributes used in the measurement of FHR and UC on CTG as available the dataset [20]. CTG results of pregnant women were classified by three experts in the field of obstetrics’ interpretations of them. This labelled dataset is comprised of recordings where and duration of labor is 30 min. We categorized our CTG set into three classes, namely: Physiological (P), Suspicious (S), and Pathological (P), as per the guidelines [20]. The data is divided into three classes based on the different CTG attributes and their profiles, as illustrated in Figure 1.

Figure 1a,b carry the depiction of scatterplot matrix for visualizing the correlation between all 21 attributes of the CTG dataset. The left side of the plot displays the scatterplots for each pair. The right side shows the Pearson correlation coefficients, while the density plot for each attribute is located on the diagonal. The Pearson correlation determines how strongly two variables are linearly correlated. A strong linear relationship is implied by values that are close to 1.

**Figure 2:**
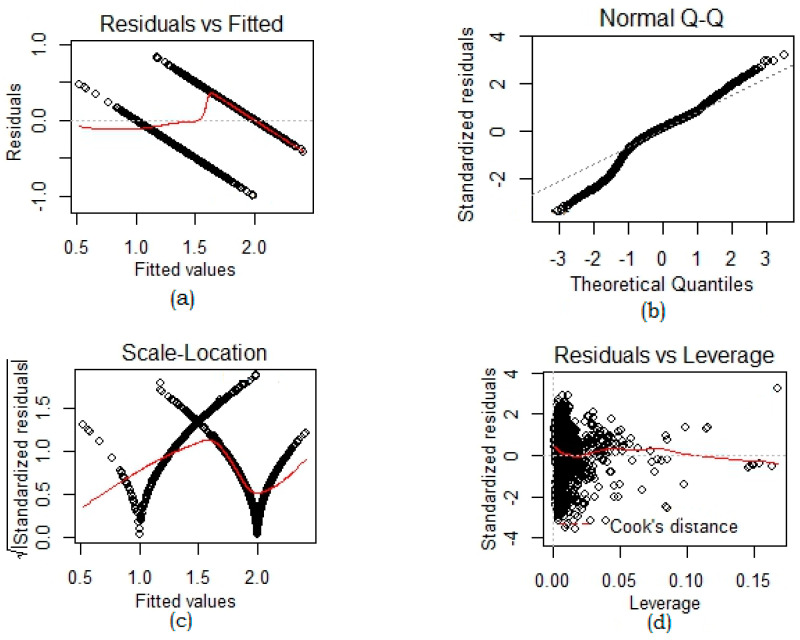
This figure is the validation of the linearity of the dataset using test plots: (**a**) indicates that there is a linear link between predictor factors and outcome variables and residuals have linear patterns; (**b**) illustrates that the residuals are normally distributed because a straight dashed line is well lined with residuals; (**c**) is the scale-location plot and confirmed that residuals are distributed evenly across the predictors’ range; (**d**) exhibits the significant data points by using Cook’s distance.

This includes LB (medical expert baseline values), fetal accelerations (AC), fetal movement (FM), percentage of time with abnormal short-term variability (ASTV), percentage of time with abnormal long-term variability (ALTV), light fetal decelerations (DL), severe fetal decelerations (DS), prolonged decelerations (DP), and repetitive decelerations (DR). Multicollinearity upturns the variance of the coefficient estimates extremely and makes the estimates very sensitive to minor changes in the model. Identifying the degree of multicollinearity in the preprocessing phase contributed to achieving the required correlation between each pair of explanatory variables. Min is the minimum of FHR histogram, Max represents the maximum of FHR; Nmax is histogram peaks; Nzeros are histogram zeros; Mode, Mean, and Median are FHR statistical data parameters. The correlation between 0.6–1.0 was considered a strong positive correlation. The topology of correlation is presented in Figure 1. Pair-wise correlation was found to be high for the pairs, including Median/Mode (0.933), Median/Mean (0.948), Min/Width (−0.899), Mean/Mode (0.893) and N_max_/Width (0.747). The purpose of this exercise is to identify the key attributes that have a strong dependence on the model performance. Using Figure 2, nine correlated attributes include: class, mean, median, mode, width, Nmax, MSTv, and variance of the CTG dataset. Since the pair-wise correlation between the explanatory variables is not a sufficient condition to determine multicollinearity, the Farrar–Glauber (FG) test was performed. The calculated value of the FG Chi-square test statistic was 33,529.57. The FG test also determined the diagnostic output for variance inflation factor (VIF) to be 26.87, in addition to the variables of mean (20.1283), min (19.6931), width (17.7735), and mode (9.0131). The results are exhibited in Figure 1.

The next process was to remove outliers using the correlations map in Figure 1. Variable predictors that have a strong dependence were excluded and the linearity of the dataset was then validated using test plots exhibited in Figure 2. Since the samples in our three classes, namely, Normal (N), Suspected (S) and Pathological (P) were imbalanced, upsampling was performed to balance the classes. We used the Imblearn library in python for data upsampling that works based on the k-nearest neighbors algorithm. We synthetically generated data points that fall in the proximity of the already existing outnumbered class. Since the sampling process is applied only to the training set, our validation and testing data remain unchanged. After balancing the data, feature scaling was performed before feeding it to our classification algorithm.

Since nonlinearity amongst different CTG parameters is important. Therefore, a linear regression analysis was performed to determine the degree of nonlinearity in our dataset, as presented in Figure 2. Residual against the fitted plot in Figure 2a indicates that the relationship between attributes in our dataset is linear as the data points are evenly spaced around the zero line and the zero line corresponds to our estimated regression line for CTG attributes. In our QQ plot, CTG data attributes represent the *y*-axis, and theoretical quantiles from a standard normal are on the *x*-axis. The middle and tails of our distribution are the same as a true normal distribution, as illustrated in Figure 2b. This helped us to validate that our data is normally distributed. Figure 2c is a spread location plot and it reflects that our residuals are evenly spread along with the range of predictor variables. The red line is horizontal across the plot, implying that the spread of CTG attributes around it is symmetric. A residual against leverage plot was performed to identify influential CTG parameters in our CTG dataset. We observed no influential points that would change our statistical distribution as presented in Figure 2d. We used Cook’s distance to impose this condition.

### 2.2. Proposed Classification Architecture

**Figure 3:**
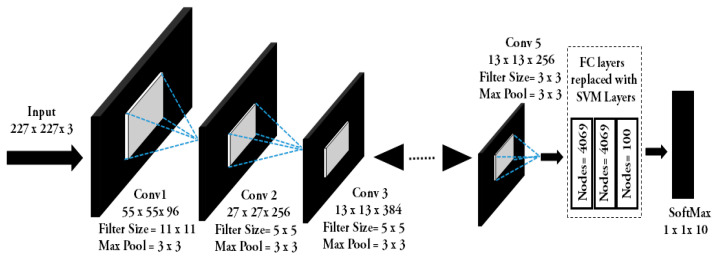
The model we employ in our dataset with the fully connected layers is replaced with SVM within the AlexNet.

We employed our newly created hybrid AlexNet-SVM architecture with an input layer, a convolution layer, a pooling layer, modified SVM fully connected layers, and an output layer. AlexNet algorithm [26,27,28,29] learns from filters in the convolution layer. The extracted features are delivered to the subsequent layer carrying multiple feature maps [29,30]. To deliver a concatenated output using max pooling or average pooling algorithms, the pooling layer concentrates on a cluster of neurons to reduce the number of weights. The dimensionality of each feature map was then reduced by downsampling it using Numpy (Python). In the pooling layers, we selected the stride, padding factor, and kernel size based on our optimization experiment. In the fully linked layer, class scores were computed. The SoftMax layer then produced a 3-dimensional vector that corresponded to the number of classes concerned. In the SoftMax classification layer as the loss function, cross-entropy was calculated [30,31]. During the training step, by setting random activations to zero, overfitting is avoided in our model by using a dropout layer followed by a fully connected layer. Figure 3 represents our proposed algorithm where input and output feature maps of each block are presented. We replaced the fully connected layers in the AlexNet with SVMs.

As illustrated in Figure 3, the cross entropy-based fully connected layers were replaced with SVMs. The training layer passed the data to the next layer when the loss function converged to zero. We classified based on the particular label vs. the rest. Our SVM layers contribute to updating the weights of all hidden layers to conserve computational time. The training process with a layer size of 25 was fed with an input tensor with a dimension of 227 × 227 × 3. The learning rate was kept initially at 0.5 along with a bias rate of 2 for low-level feature learning. Data transference was achieved and the learning rate at the fully connected layer was set to a higher value of 17 to enable the network to learn high-level abstract features in a smaller span of time from the pretrained layers. For model training, we used the Adam optimizer. It computes individual adaptive learning rates for different parameters from estimates of first and second moments of gradients. The learning rate for initial layers was set to 0.3 and for end layers, it was L_r_ = 10^−4^. The exponential decay rates (β_1_ and β_2_) of the first and second moment estimates were 0.9 and 0.99, respectively, with Є = 1 × 10^−8^. To find the optimal solution in minimal time, we reduced the learning rate by a factor of two when the validation error saturated. The algorithm for our newly created model is presented in Algorithm 1.
**Algorithm 1** Function AlexNet-SVM (A, T, W).1: Input: AlexNet Model: A, Kernel Dimensions: Ki,2: Pre-trained weights of individual layers: [w_1_, w_2_,…,w_n_]3: Output: SVM Merged AlexNet Model: Asvm,4: Define model parameters     # classifier, bias, optimizer5: **For** i ← 1 to Layers **do**       # classes in data load6: **if** layers = = Conv then7: Min Batch = 10;          # Minimum Batch Size8: Learning Rate = 1 × 10^−4^;9: output= AlexNet (data)10: loss ← cross_entropy (output, classes)   # Loss Calculation11: optimizer. zero_grad ();         # Update weights10: loss, Backward ();12: **end**13: LT= ← net.Layers (1:end-3)   # Replacing Fully Connected Layers (FCL)14: Layers = LT, FCL (3, LF’20, b’20));      # LT (Layer Transfer), LF (Learn factor)15: SVM_L ← concatenate ((train_L), (validate_L));  # SVM_L (SVM Label)16: Asvm = (A, Wm, FCL)          # Wm (modified weights of layers)17: **end**

As illustrated in Algorithm 1, the number of convolution layers is equal to the convolution operations to be performed. Our model considers an input dimensional feature of 227 × 227 × 3. Then we apply the first convolution layer with 96 filters of size 11 × 11 with a stride of 4. The output feature map is 55 × 55 × 96. Next, we apply max pooling and produce the resulting feature map with the size of 27 × 27 × 96. After this, we apply the second convolution operation with 256 filters to obtain an output size of 27 × 27 × 256. Then we receive a max-pooling layer of size 3 × 3 with stride 2 and the resulting feature map becomes size 13 × 13 × 256. Similarly, after applying third and fourth convolution layers the feature map, the dimensions remained 13 × 13 × 384. The mini-batch size remained at 10 during these operations, as indicated in Algorithm 1. The final convolutional layer has a feature map of 13 × 13 × 256. The learning rate was set to 1 × 10^−4^. The loss was computed and backpropagated to update the layer weights. We replaced the final three layers with SVMs in our model and used cross-entropy to converge the loss function by updating the weights of layers. This concluded in an AlexNet-SVM merged model.

**Figure 4:**
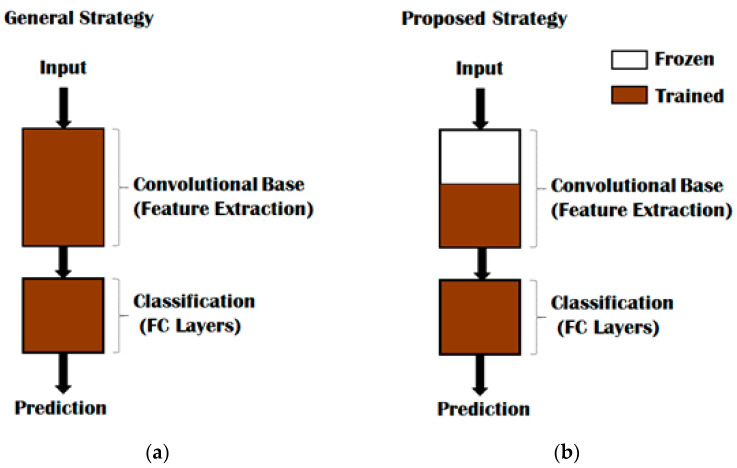
Training of the DNN Model: (**a**) indicates a general strategy where both convolutional base and classification layers are trained; (**b**) represents our proposed strategy where we froze part of the layers in the convolutional base.

Transfer learning is a key aspect to improving the learning in the target domain, and overparametrization was avoided by sophisticated feature reuse through data clustering [32,33,34,35]. The labelled data was classified by setting up feature spaces based on their marginal probabilities. This architecture enabled us to capture different features at different levels in the network. Typically, any DNN has two parts: a convolutional base that is composed of convolution and pooling layers for general feature learning, and a classifier that is usually composed of fully connected layers. Rather than following a general strategy as indicated in Figure 4a, where the training of the entire model is done based on the dataset, we employed an optimum strategy as illustrated in Figure 4b to preserve computational time.

Transfer learning is implemented by leveraging the generic features for labels that are available in both the source and target domains. Feature extraction was performed using AlexNet, in which FHR signals are passed through a set of preprocessing procedures. When these generalized features were acquired in layers, we removed the fully connected layers and added lightweight SVM, as indicated in Figure 4b. Then we trained the newly added connected layers for specific learning tasks by freezing the weights of the earlier layers. Freezing the layers allows us to keep the learned data intact through transfer learning during the training phase of top layers. After the top layers were trained, we performed fine-tuning to complete the transfer learning phase. We normalized our network predictions based on the cross-entropy (CE) between the true label distribution and the predicted label using Equation (1).

(1) $$E=-\frac{1}{N}\sum_{i}y_{i}\log\left(Y_{i}\right)$$

where −1/*N* represents the number of samples, *y*_i_ is the true label, and *Y*_i_ indicates our predicted label.

Cross-entropy was utilized to help predict an outcome compared to the true outcome. The use of a negative algorithmic function allows us to retrieve the error function for each data point to determine the predicted label as compared to the true label. 

### 2.3. Performance Evaluation of Proposed Classification Architecture

For the evaluation of our proposed algorithm, several metrics, including accuracy, precision, and recall were utilized as defined in Equations (2)–(4).

(2) $$Accuracy=\frac{TP+TN}{TP+TN+FP+FN}$$

(3) $$Accuracy=\frac{TP+TN}{TP+TN+FP+FN}$$

(4) $$Recall=\frac{TP}{TP+FN}\times 100\%$$

where *TP* represents True Positive, *TN* is True Negative, *FP* is False Positive, and *FN* indicates False Negative.

For multiclass classification results on each model and their intercomparison with our proposed model accuracy, precision and recall were calculated. Accuracy captured the percentage of correct predictions of overall test data, where precision and recall measured the ability of a model to identify relevant data points within a dataset.

A key performance factor for our proposed algorithm is computational efficiency in clinical settings. To establish the computational performance, processing time, system time, and elapsed times for different leading algorithms for our dataset were measured and compared to our proposed algorithm. We defined processing time as a combination of forwarding propagation, backward propagation, and update time for each layer. We split DNNs into different layers with the fully connected layer as a special convolutional layer. The algorithm for the processing time is exhibited in Algorithm 2. Elapsed time refers to the time taken by the CPU to compute the expressions. This is an aggregate of the user and system time. User time is the time taken by the CPU to execute the code, whereas the system time is the user time plus the time taken to compute the kernel function. We defined the parameters a and b, in addition to vector values that are randomly sampled for time computations. The function proc.time () determined the processing time. We started with a vector of 100,000, and this value was replaced against the constant in Algorithm 2. The function proc.time () works as a stopwatch, and we initialized it to a starting time. Then we added 1 to each of these values and ran our code. Subtracting the starting time from the ending time provided the processing time of our developed model, as illustrated in Algorithm 2.
**Algorithm 2** Computation of Processing Time.1: Input: Model Parameters: Mp, DNN Architecture: Da,2: Output: Results for Processing time3: Define Parameters.4: a normal (constant)5: b  rep(NA, constant)     #replicate numerical values6: **For** each layer L belongs to [1, N] **do**7: Pt proc. time ();     #Start the clock8: **end**9: For (i == constant){10: b [i]  a [i]+1;11: }12: proc. time ()-Pt;     # Stop the clock13: output = Pt

## 3. Results

**Figure 5:**
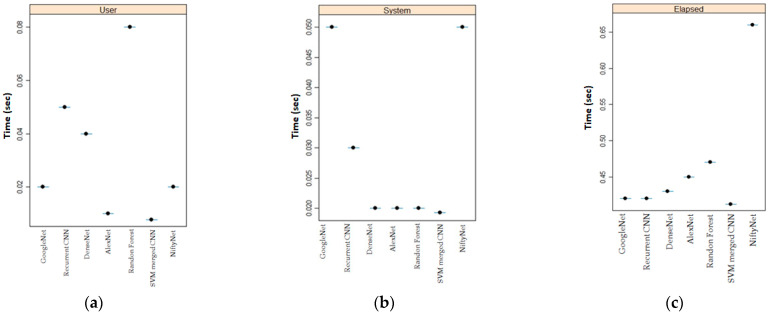
An intercomparison of time complexity for different classification algorithms.

A train validation-test strategy was used in all the studies discussed in this section. On the test set, the provided results were computed while the selection of hyperparameters was made over the validation set. We implemented and tested all leading deep neural algorithms for our CTG dataset. These include: recurrent neural networks, random forests, GoogleNet, DesnseNet, NiftyNet, AlexNet, and our proposed SVM AlexNet. As indicated in Figure 5a–c, our proposed algorithm performed best with respect to time complexity for user, system and elapsed scenario. Time complexity was gauged in terms of elapsed time, user time, and system time. Our SVM AlexNet hybrid classification architecture resulted in faster convergence by avoiding weight recalculation in all layers. Contrary to our presented method where resources are only spent on determining the global gradient, leading reported architectures require intense time and space resources to compute local maxima [32,33,34,35,36,37,38].

**Figure 6:**
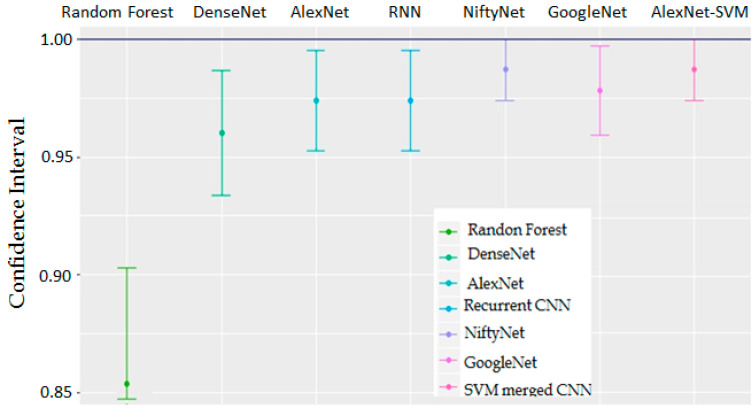
An intercomparison of the proposed method with the leading architectures using 95% CI.

In AlexNet, the proportion of fully connected and convolution layers is more than 90% as compared to other algorithms. It can be observed that the prediction accuracy of our model for the fully connected layer is significantly better than the state-of-the-art algorithms presented in Figure 6. Parameter optimization was performed according to the validation set with the convolution kernel of size 5 × 5. It has been revealed that when the mini-batch size was equal to 64, the validation accuracy remained higher, regardless of the max epoch. Conversely, it was observed that the model 10 epochs required the input data to learn the maximum possible features of the fetal state.

To improve the efficiency of the training of the feed-forward neural network, we used the ADAM optimizer for the backpropagation. After our proposed DNN algorithm is trained, it immediately starts classifying an unidentified fetus within our three label classes. The primary advantage of our proposed method is based on the deep architecture’s convolutional layers, which provide discrete local features to characterize the input data. 

To make our findings clinically significant, we calculated a confidence interval (CI) for each sample. This implies that we anticipate our dataset mean to be found within 95% of these CIs. We compared our proposed algorithm with the commercially available algorithms, including GoogleNet, DenseNet, and NiftyNet, on the basis of CI. Figure 6 indicates that the 95% CI of our algorithm is where the population parameter is likely to reside, while CI computations, sample variability, and sample sizes were kept constant for all algorithms.

Table 1 illustrates the specificity, sensitivity, and balanced accuracy indices of the proposed algorithm against the leading methods for our chosen dataset. Our presented classification architecture outperformed the other methods on the same CTG dataset in accuracy for both the Suspected and Pathological fetal recordings.

## 4. Discussion

### 4.1. Merged (AlexNet-SVM) Architecture

Data visualization and the correlation analysis discovered that AC, FM, ASTV, ALTV, DL, DS, DP, and DR were the most relevant attributes for the fetal-state evaluation. Our model supplemented the CTG construal rules in the fetus classification. Our regressive analysis revealed that variability could predict baseline and uterine activity could predict deceleration movements of the fetus; therefore, fetus baseline, acceleration, and deceleration variables are essential for a potential clinical decision support system. Figure 1 illustrated the degree of multicollinearity between each pair of fetus-state variables. DNNs receive inputs, perform a dot operation, and then express the mapping between test data to their respected labels. Our model is a very effective way to use DNN as a feature extractor, and to to SVM with these features to predict a label, resulting in a better time and classification performance. The high-end layer was substituted by SVM to prevent our network from overfitting dropout is applied. Compared to our algorithm’s accuracy of 99.72%, the classification accuracy of SVM, multi-layer perceptron (MLP), and DNN remained at 79.66%, 85.98%, and 93.24%, respectively [18]. Similarly, in a previous study [23], the following 10 pretrained fetus networks: Alexnet, Vgg16, Vgg19, Squeezenet, Googlenet, Inception v3, Densenet 201, Resnet 18, Resnet 50, and Resnet 101 recorded the highest classification accuracy of 82.85%. Even smaller datasets of CTG recordings reported accuracy between 80% to 93% [27,31].

### 4.2. Transfer Learning

To achieve multiclassification for our imbalanced CTG dataset and heavy convolution layers involved in AlexNet, we used transfer learning. Transfer learning allowed us to skip weight recalculations and upgradations for frozen layers. Figure 4 and Figure 5 and Algorithm 2 indicate that there is a significant difference in time performance between training from scratch and fine-tuning. Using grid search, our proposed model removed the fully connected layers to achieve a less time-intensive solution. This is predominantly useful for our model with heavy convolutional layers. The pooling layers ensure overlapping amongst local receptive fields, hence minimizing the error in our model. We extracted all layers except the last three. For transfer learning, we set the learning rate high for the new layers and slower for the older ones by specifying the mini-batch size and validation data. Fine-tuning after transfer learning is less expensive compared to fine-tuning from scratch learning due to partial updates of the parameters of the convolutional layers. Our transfer learning task achieved the desired results when tied to pretrained models.

### 4.3. Computational Complexity and Classification Accuracy

Another key contribution of the presented work is the reduced computational complexity. This key parameter for real-time settings has never been given due attention in the reported literature for fetus classification. MLOps suggest that every evolved model must be gauged under a limited computational budget for real-time implementations. This motivates a series of works toward a speed–accuracy tradeoff using lightweight architectures [33]. It has been reported that DNNs achieve classification accuracy between 84–93% at the cost of intense computational effort that ranges from 118.90 s to 1330 s [33,34]. In [35], random forests were used for the classification of the same dataset with achieved accuracy of 93.6%. The intense computational nature of this model hindered its automation for fetus classification. SVMs provide reasonable accuracy but they are not preferred for large CTG datasets, as the complexity of the algorithm’s training is a direct function of the dataset size [36]. DNNs [37] can be trained with a high-dimensional CTG dataset but excessive connections severely decrease computational efficiency, as reported previously [38]. In [38,41], DenseNet is reported to exploit dense concatenation blocks for feature mapping, but the heavy processing time makes them unsuitable for clinical settings. Multilayer perceptron and long short-term memory (LSTM) networks [39,40] are characterized by several layers of input nodes connected as a directed graph with the output. They both include a very dense web of parameters, resulting in redundancy and inefficiency. An intercomparison of all these models with our proposed model on the CTG dataset with respect to computational efficiency is presented in Figure 5. Our proposed model outperformed the leading architectures in processing time by merging a lightweight SVM at the high-end layers. Our proposed model utilizes broader temporal information to extract CTG features and integrate them at higher layers using lightweight SVMs for classification. Since we have imbalanced classes, we presented an intercomparison between our model and other cutting classification architectures. Our model outpaced the classification accuracy, sensitivity, and specificity of cutting-edge models.

## 5. Conclusions

The translational fusion of deep learning algorithms with CTG data resulted in promising results in terms of time computations and classification accuracy. We achieved better time-performance results that are needed for clinical time settings. Our algorithm outperformed the leading architectures with an accuracy of 99.72%, sensitivity of 96.67%, and classification specificity of 99.6%. Compared to the cutting-edge algorithms, our model resulted in a more local objective function. The incorporation of our model to predict compromised fetuses would enable timely referral and informed decision-making in clinical practice.

## Figures and Tables

**Table 1:** Performance indices of the proposed method routine with other leading methods on the same CTG dataset.

Statistics by Class	“Sensitivity”	“Specificity”	“Pos Pred Value”	“Neg Pred Value”	“Prevalence”	“Detection Rate”	“Detection Prevalence”	“Balanced Accuracy”
Total number of observations: 2126				Normal recordings: 1655				
Random Forest [35]	0.9029	0.7632	0.9461	0.6304	0.8216	0.7418	0.7840	0.8330
LS-SVM [36]	0.8128	0.8000	0.9811	0.1739	0.9531	0.7746	0.7840	0.8064
AlexNet [37]	0.9866	0.6875	0.8802	0.9565	0.6995	0.6901	0.784	0.8370
DenseNet [38]	0.8445	0.8236	0.8653	0.8245	0.784	0.784	0.784	0.9367
MLP [39]	0.8394	0.8289	0.8199	0.8083	0.6887	0.8840	0.8840	0.8598
LSTM [40]	0.9744	0.9621	0.9534	0.921	0.833	0.925	0.8870	0.9625
CWT-CNN [41]	0.9012	0.8721	0.8981	0.9873	0.756	0.756	0.757	0.9408
Proposed architecture	0.9894	0.9877	0.9982	0.9925	0.784	0.784	0.784	0.9991
Pathological recordings: 176								
Random Forest [35]	0.8628	0.95610	0.47059	1.000	0.03756	0.03756	0.07981	0.8780
LS-SVM [36]	0.8888	0.95588	0.47059	0.9949	0.04225	0.03756	0.07981	0.9023
AlexNet [37]	0.9232	0.96552	0.58824	1.000	0.04695	0.04695	0.07981	0.8927
Densenet [38]	0.9161	0.98492	0.82353	1.000	0.06573	0.06573	0.07981	0.8724
MLP [39]	0.9411	0.98000	0.76471	1.000	0.06103	0.06103	0.07981	0.9151
LSTM [40]	0.9652	0.9634	0.7921	1.000	0.06521	0.0671	0.07981	0.9210
CWT-CNN [41]	0.9753	0.9843	0.8322	1.000	0.07412	0.0667	0.07981	0.9523
Proposed architecture	1.000	0.99492	0.94118	1.000	0.07512	0.07512	0.07981	0.9974
Suspect recordings: 295								
Random Forest [35]	0.5000	0.9235	0.5172	0.91848	0.14085	0.07042	0.13615	0.7117
LS-SVM [36]	0.7200	0.9816	0.8966	0.8696	0.2347	0.1221	0.1221	0.8608
AlexNet [37]	0.8056	0.9783	0.8566	0.9620	0.1690	0.1362	0.1362	0.9028
DenseNet [38]	0.9032	0.9545	0.9615	0.9837	0.1455	0.1315	0.1362	0.8889
MLP [39]	0.8788	0.9655	0.9834	0.9783	0.1549	0.1362	0.1362	0.9194
LSTM [40]	0.8921	0.9678	0.9873	0.9838	0.1564	0.1362	0.1315	0.9675
CWT-CNN [41]	0.9512	0.985	0.9887	0.9765	0.1456	0.1362	0.1362	0.9876
Proposed architecture	0.9667	0.996	1.0000	0.9946	0.1408	0.1362	0.1362	0.9972

## Data Availability

The CTG dataset used in our study is publicly available at UCI Machine Learning Repository: Cardiotocography Data Set at https://archive.ics.uci.edu/ml/datasets/cardiotocography (accessed on 22 January 2022).

## Abbreviations

AC	Fetal Accelerations
AI	Artificial Intelligence
ALTV	Abnormal Long-Term Variability
ANNs	Artificial Neural Networks
ASTV	Abnormal Short-Term Variability
CE	Cross-Entropy
CI	Confidence Interval
CNNs	Convolutional Neural Networks
CTG	Cardiotocographic
CTGs	Cardiotocograms
DL	Light Fetal Decelerations
DNNs	Deep Neural Networks
DP	Prolonged Decelerations
DR	Repetitive Decelerations
DS	Severe Fetal Decelerations
ELNs	Extreme Learning Networks
FG	Farrar Glauber
FHR	Fetal Heart Rate
FM	Fetal Movement
LB	Baseline
LSTM	Long Short-Term Memory
LSTMs	Long Short-Term Memory Networks
ML	Machine Learning
MLOps	Machine Learning Operations
MLP	Multilayer Perceptron
N	Normal
P	Pathological, Physiological
S	Suspicious, Suspected
SVMs	Support Vector Machines
TL	Transfer Learning
UC	Uterine Contraction
VIF	Variance Inflation Factor
